# Compatible extension of the $\left(G^{\prime }/G\right)$-expansion approach for equations with conformable derivative

**DOI:** 10.1016/j.heliyon.2023.e15717

**Published:** 2023-04-26

**Authors:** Altaf A. Al-Shawba, Farah A. Abdullah, Amirah Azmi, M. Ali Akbar, Kottakkaran Sooppy Nisar

**Affiliations:** a School of Mathematical Sciences, Universiti Sains Malaysia, Malaysia; b Department of Applied Mathematics, University of Rajshahi, Bangladesh; c Department of Mathematics, College of Science and Humanities in Alkharj, Prince Sattam bin Abdulaziz University, Alkharj, Saudi Arabia; d School of Technology, Woxsen University, Hyderabad, 502345, Telangana State, India

**Keywords:** Fractional coupled burgers equations, Fractional derivative, (*G’/G*)-Expansion approach, Soliton solutions

## Abstract

In this study, the compatible extensions of the $\left(G^{\prime }/G\right)$-expansion approach and the generalized $\left(G^{\prime }/G\right)$-expansion scheme are proposed to generate scores of radical closed-form solutions of nonlinear fractional evolution equations. The originality and improvements of the extensions are confirmed by their application to the fractional space-time paired Burgers equations. The application of the proposed extensions highlights their effectiveness by providing dissimilar solutions for assorted physical forms in nonlinear science. In order to explain some of the wave solutions geometrically, we represent them as two- and three-dimensional graphs. The results demonstrate that the techniques presented in this study are effective and straightforward ways to address a variety of equations in mathematical physics with conformable derivative.

## 1 Introduction

In contemporary years, nonlinear fractional evolution equations (NFEEs) have appealed the attention of the researchers for their importance in describing the internal processes of real-world incidents. NFEEs appear in various technical and industrial arenas, for instance, plasma physic, electricity, fluid mechanics, modelling of earthquake, quantum physics, chemical kinematics, water wave mechanics, etc. Traveling-wave solutions of NFEEs are crucial for understanding the underlying workings of complicated physical processes. Thus, several methods for extracting explicit wave solutions have been developed by researchers. For example, the inverse scattering approach [1], the Hirota's bilinear transformation [2], the Painleve expansion in truncated type [3], the Jacobi elliptic function approach [4,5], the Weierstrass elliptic function scheme [6], the fractional Riccati equation approach of the Bäcklund transformation [7], exponential-function technique [8,9], tanh-function approach [10,11], the systematic approach [12], and others [13].

In order to attain diverse exact solutions to some NLEEs, Wang et al. [14] presented an important new approach, called the $\left(G^{\prime }/G\right)$-expansion approach, where G(ξ) satisfies the linear differential equation: $G^{\prime \prime }+\lambda G^{\prime }+\mu G=0$; μ and λ are subjective parameters. For better understanding of the $\left(G^{\prime }/G\right)$-expansion approach, the applications of this method in NFEEs can be found in Refs. [[15], [16], [17], [18], [19], [20], [21], [22]].

Diverse academics have conducted further research to demonstrate the validity and efficacy of the $\left(G^{\prime }/G\right)$-expansion approach and to widen its scope of applicability; such as, Zhang et al. [23] suggested the improved $\left(G^{\prime }/G\right)$-expansion scheme. In the elementary method, the solution was offered in the shape $u\left(\xi \right)=\sum_{j=0}^{n}a_{j}{\left(G^{\prime }/G\right)}^{j}$, where $a_{n}\neq 0$, but in Ref. [23] Zhang et al. used $u\left(\xi \right)=\sum_{j=-n}^{n}a_{j}{\left(G^{\prime }/G\right)}^{j}$ as a solution. Many researchers studied various NFEEs to determine exact solutions expending the improved $\left(G^{\prime }/G\right)$-expansion method which can be found in Refs. [[24], [25], [26], [27]]. Guo and Zhou [28] suggested the extended $\left(G^{\prime }/G\right)$-expansion approach: $u\left(\xi \right)=a_{0}+\sum_{j=1}^{n}\{a_{j}\;{\left(G^{\prime }/G\right)}^{j}$ $+b_{j}\;{\left(G^{\prime }/G\right)}^{j-1}\;\sqrt{\sigma \left(1+{\left(G^{\prime }/G\right)}^{2}/\mu \right)}\}$ and investigated exact solutions to the Whitham-Broer-Kaup-like equation and coupled Hirota-Satsuma KdV equations. Later, different researchers applied this extended approach to construct different classes of traveling wave solutions for some NFEEs [29,30]. In order to obtain exact results of the KdV equation, the stress equation for micro-structured objects and the ZKBBM equation, Akbar et al. [31] suggested a comprehensive and enhanced $\left(G^{\prime }/G\right)$-expansion methodology, wherein the solution is offered in the formula $u\left(\xi \right)=\sum_{j=-n}^{n}\frac{c_{-j}}{{\left(d+\left(G^{\prime }/G\right)\right)}^{j}}$, where together $c_{-n}$ and $c_{n}$ should not be zero consecutively. Naher and Abdullah [32] proposed the generalized $\left(G^{\prime }/G\right)$-expansion process in which the result was offered by the formula $u\left(\xi \right)=\sum_{j=0}^{n}a_{j}{\left(d+G^{\prime }/G\right)}^{j}+\sum_{j=1}^{n}b_{j}{\left(d+G^{\prime }/G\right)}^{-j}$, where $a_{j}$, $b_{j}$, d are parameters and they obtained new exact solutions for KdV equation.

Since each nonlinear fractional equation has its specific meaningful rich structure, there is still a lot of work to be done before the original $\left(G^{\prime }/G\right)$-expansion approach can be fully established. Therefore, in this investigation, we suggest a development of the $\left(G^{\prime }/G\right)$-expansion approach and the generalized $\left(G^{\prime }/G\right)$-expansion approach in the case of the conformable fractional derivative suitable for searching NFEEs. To validate the novelty and suitability of the proposed extensions, the space-time fractional CB equations are exposed and assorted descriptive traveling wave solutions are uncovered.

## 2 The conformable fractional derivative

The β-order conformable derivative of a function f is defined as [[33], [34], [35]]:

$$T_{\beta }\left(f\right)\left(t\right)=\lim_{h\to 0}\frac{f\left(t+h\;t^{1-\beta }\right)-f\left(t\right)}{h}\text{,}$$

where f:[0,∞[→R, t>0 and β∈(0,1].

Suppose f and g be β-differentiable at the point x, then some properties for $T_{\beta }$ are listed below:

(i) $T_{\beta }\left(cf+eg\right)=cT_{\beta }\left(f\right)+eT_{\beta }\left(g\right),\forall c,e\in R$.
(ii) $T_{\beta }\left(x^{\mu }\right)=\mu x^{\mu -\beta },\forall \mu \in R$.
(iii) $T_{\beta }\left(fοg\right)\left(x\right)=x^{1-\beta }g'\left(x\right)f'\left(g\left(x\right)\right)$.
(iv) $T_{\beta }\left(fg\right)\left(x\right)=fT_{\beta }\left(g\right)+gT_{\beta }\left(f\right)$.
(v) $T_{\beta }\left(\frac{f}{g}\right)\left(x\right)=\frac{gT_{\beta }\left(f\right)-fT_{\beta }\left(g\right)}{g^{2}}$.

Since f is a differentiable function, then $T_{\beta }\left(f\right)\left(x\right)=x^{1-\beta }\frac{df}{dx}\left(x\right)$.

## 3 Algorithm of the extensions

Let us assign a general NFEE of the shape:

(1) $$Q\left(u,D_{t}^{\alpha }u,D_{x}^{\beta }u,D_{y}^{\gamma }u,D_{z}^{\delta }u,D_{t}^{2\alpha },D_{t}^{\alpha }D_{x}^{\beta }u,D_{x}^{\beta }D_{x}^{\beta }u,\ldots \right)=0,0<\alpha ,\beta ,\gamma ,\delta \leq 1\text{,}$$

where $D_{t}^{\alpha }u,D_{x}^{\beta }u,D_{y}^{\gamma }u$ and $D_{z}^{\delta }u$ indicate conformable derivatives of the wave function u relating to x,y,z and t.

Step 1 Combine the temporal variable t and spatial variables x,y and z by the wave coordinate ξ as:

(2) $$u\left(x,y,z,t\right)=u\left(\xi \right),\xi =K\frac{x^{\beta }}{\beta }+N\frac{y^{\gamma }}{\gamma }+M\frac{z^{\delta }}{\delta }\pm C\frac{t^{\alpha }}{\alpha }\text{,}$$

where K,N and M are non-zero constraints and C is the wave transmission rate. Eq. (1) is transformed by the fractional wave conversion Eq. (2) into a nonlinear equation of classical derivative for u=u(ξ):

(3) $$F\left(u,u^{\prime },u^{\prime \prime },u^{\prime \prime \prime },\ldots \right)=0$$

where F is a nonlinear function of u(ξ) and its total derivatives.

Step 2 Eq. (3) will be integrated based on the possibilities.

### 3.1 Extension of the $\left(G^{\prime }/G\right)$-expansion method

Step 3 Assume that using the presented approach, the estimation of Eq. (3) is given ensuing:

(4) $$u\left(\xi \right)=\sum_{j=0}^{n}a_{j}{\left(d+1/\varphi \right)}^{j}+\sum_{j=1}^{n}b_{j}{\left(d+1/\varphi \right)}^{-j}$$

where any one of $a_{n}$ or $b_{n}$ might be vanished, $a_{j}\left(j=0,1,2,\ldots ,n\right)$, $b_{j}\left(j=1,2,3,\ldots ,n\right)$ and d are subjective parameters to be calculated afterward, and φ(ξ) is specified below:

(5) $$\varphi \left(\xi \right)=\left(G^{\prime }/G\right)$$

where G=G(ξ) fulfills the subsequent second-order equation:

(6) $$G^{\prime \prime }+\lambda G^{\prime }+\mu G=0$$

where λ and μ are constraints.

Step 4 The assessment of n in Eq. (4) can be obtained by applying the homogeneous balancing principle between the maximal-order derivative and nonlinear extents in equation (3).

It is obtained following three solutions of equation (5) expending the generalized estimations of equation (6):

**Family 1**: When $\rho =\lambda^{2}-4\mu >0$,

(7) $$\varphi \left(\xi \right)=\left(G^{\prime }/G\right)=\frac{\sqrt{\rho }}{2}\left\{\frac{\sinh\left(\xi \frac{\sqrt{\rho }}{2}\right)A_{1}+\cosh\left(\xi \frac{\sqrt{\rho }}{2}\right)A_{2}}{\cosh\left(\xi \frac{\sqrt{\rho }}{2}\right)A_{1}+\sinh\left(\xi \frac{\sqrt{\rho }}{2}\right)A_{2}}\right\}-\frac{\lambda }{2}\text{,}$$

where $A_{1}$ and $A_{2}$ are subjective constants.

**Family 2**: When $\rho =\lambda^{2}-4\mu <0$,

(8) $$\varphi \left(\xi \right)=\left(G^{\prime }/G\right)=\frac{\sqrt{-\rho }}{2}\left\{\frac{-\sin\left(\frac{\sqrt{-\rho }}{2}\;\xi \right)A_{1}+\cos\left(\frac{\sqrt{-\rho }}{2}\;\xi \right)A_{2}}{\cos\left(\frac{\sqrt{-\rho }}{2}\;\xi \right)A_{1}+\sin\left(\frac{\sqrt{-\rho }}{2}\;\xi \right)\;A_{2}}\right\}-\frac{\lambda }{2}$$

**Family 3**: When $\rho =\lambda^{2}-4\mu =0$,

(9) $$\varphi \left(\xi \right)=\left(G^{\prime }/G\right)=\frac{A_{2}}{A_{1}+A_{2}\xi }-\frac{\lambda }{2}$$

Step 5 Replacing solution (4) together with Eqs. (6), (5), (3) with the assessment of n gained in Step 4, gives polynomial of ${\left(d+1/\varphi \right)}^{n},\left(n=0,1,2,\ldots \right)$ and ${\left(d+1/\varphi \right)}^{-n},\left(n=1,2,3,\ldots \right)$. Then, collecting each cohort of the ensued polynomial to zero, provides a group of arithmetical equations for $a_{j}\left(j=0,1,2,\ldots ,n\right),b_{j}\left(j=1,2,\ldots ,n\right),d,\lambda ,\mu ,K\text{,}$ N,M and C.

Step 6 Assume that the assessment of the coefficients $a_{j}\left(j=0,1,2,\ldots ,n\right),b_{j}\left(j=1,2,3,\ldots ,n\right),d,K\text{,}$ N,M and C can be gained by resolving the equations ascertained in Step 5. The constant measures, along with the generic estimations of (6), are then substituted into Eq. (4), yielding new general type and expedient solutions of the NFEE (1).

### 3.2 Extension of the generalized $\left(G^{\prime }/G\right)$-expansion method

Step 7 As per this technique the solution of Eq. (3) is presented in the form:

(10) $$u\left(\xi \right)=\sum_{j=0}^{n}a_{j}{\left(d+1/\varphi \right)}^{j}+\sum_{j=1}^{n}b_{j}{\left(d+1/\varphi \right)}^{-j}\text{,}$$

where either $a_{n}$ or $b_{n}$ might be zero, $a_{j}\left(j=0,1,2,\ldots ,n\right)$, $b_{j}\left(j=1,2,3,\ldots ,n\right)$ and d are parameters to be computed well along, and φ(ξ) is in the form:

(11) $$\varphi \left(\xi \right)=\left(G^{\prime }/G\right)\text{,}$$

where G=G(ξ) satiates the ensuing second-order nonlinear equation:

(12) $$AGG^{\prime \prime }-BGG^{\prime }-EG^{2}-D{\left(G^{\prime }\right)}^{2}=0\text{,}$$

where A,B,E,D are constraints and prime denotes the derivative relating to ξ.

Step 8 It is ascertained that the integer n can be found from Step 4. It can be obtained the resulting five solutions of Eq. (11) by means of the generalized solutions of Eq. (12).

**Family 1**: If B≠0, $\Omega =B^{2}+4E\left(A-D\right)>0$ and Φ=A−D,

(13) $$\varphi \left(\xi \right)=\left(G^{\prime }/G\right)=\frac{\sqrt{\Omega }}{2\Phi }\left(\frac{A_{1}\;\sinh\left(\xi \frac{\sqrt{\Omega }}{2A}\right)+A_{2}\;\cosh\left(\xi \frac{\sqrt{\Omega }}{2A}\right)}{A_{1}\;\cosh\left(\xi \frac{\sqrt{\Omega }}{2A}\right)+A_{2}\;\sinh\left(\xi \frac{\sqrt{\Omega }}{2A}\right)}\right)+\frac{B}{2\Phi }$$

where $A_{1}$ and $A_{2}$ are integral constants.

**Family 2**: If $B\neq 0,\Omega =B^{2}+4E\left(A-D\right)<0$ and Φ=A−D,

(14) $$\varphi \left(\xi \right)=\left(G^{\prime }/G\right)=\frac{\sqrt{-\Omega }}{2\Phi }\;\left(\frac{{-A}_{1}\;\sin\left(\frac{\sqrt{-\Omega }}{2A}\;\xi \right)+A_{2}\;\cos\left(\frac{\sqrt{-\Omega }}{2A}\;\xi \right)}{A_{1}\;\cos\left(\frac{\sqrt{-\Omega }}{2A}\;\xi \right)+A_{2}\;\sin\left(\frac{\sqrt{-\Omega }}{2A}\;\xi \right)}\right)+\frac{B}{2\Phi }$$

**Family 3**: If $B\neq 0,\Omega =B^{2}+4E\left(A-D\right)=0$ and Φ=A−D,

(15) $$\varphi \left(\xi \right)=\left(G^{\prime }\left(\xi \right)/G\left(\xi \right)\right)=\frac{A_{2}}{{A_{2}\;\xi +A}_{1}}+\frac{B}{2\Phi }$$

**Family 4**: If B=0,Δ=EΦ>0 and Φ=A−D,

(16) $$\varphi \left(\xi \right)=\left(G^{\prime }/G\right)=\frac{\sqrt{\Delta }}{\Phi }\;\frac{\cosh\left(\frac{\sqrt{\Delta }}{A}\xi \right)A_{1}+\sinh\left(\frac{\sqrt{\Delta }}{A}\xi \right)A_{2}}{\sinh\left(\frac{\sqrt{\Delta }}{A}\xi \right)\;A_{1}+\cosh\left(\frac{\sqrt{\Delta }}{A}\xi \right)A_{2}}$$

**Family 5**: If B=0,Δ=EΦ<0 and Φ=A−D,

(17) $$\varphi \left(\xi \right)=\left(G^{\prime }/G\right)=\frac{\sqrt{-\Delta }}{\Phi }\;\frac{-A_{1}\;\cos\left(\xi \frac{\sqrt{-\Delta }}{A}\right)+A_{2}\;\sin\left(\xi \frac{\sqrt{-\Delta }}{A}\right)}{A_{1}\;\sin\left(\xi \frac{\sqrt{-\Delta }}{A}\right)+A_{2}\;\cos\left(\xi \frac{\sqrt{-\Delta }}{A}\right)}\text{.}$$

Step 9 Replacing solution (10) together with (12), (11), (3) with the estimation of n gained in Step 8, yield polynomials in ${\left(d+1/\varphi \right)}^{n}$, (n=0,1,2,…) and ${\left(d+1/\varphi \right)}^{-n}$, (n=1,2,…). Collecting each cohort of the ensued polynomials to zero, yields a set of algebraic equations for $a_{j}\left(j=0,1,2,\ldots ,n\right)$, $b_{j}\left(j=1,2,3,\ldots ,n\right)$, d, K, N, M and C.

Step 10 Assume that the assessment of parameters $a_{j}\left(j=0,1,2,\ldots ,n\right)$, $b_{j}\left(j=1,2,3,\ldots ,n\right)$, d, K, N, M and C can be obtained by unravelling the algebraic equations found in Step 9. Afterward, substituting the constant values together with the general solutions of Eq. (12), (10) yields more broad-spectrum solutions and fresh solutions of the NFEE (1).

## 4 Application of the suggested extensions

In this segment, we put on the suggested extensions to examine the coupled time-space fractional CB equations. The time-space fractional CB equation [17,36] is:

(18) $$D_{t}^{\alpha }u-D_{x}^{2\alpha }u+2uD_{x}^{\alpha }u+pD_{x}^{\alpha }\left(uv\right)=0\text{,}$$

(19) $$D_{t}^{\alpha }v-D_{x}^{2\alpha }v+2vD_{x}^{\alpha }v+qD_{x}^{\alpha }\left(uv\right)=0\text{,}$$

where 0<α<1 and the system parameters, such as Brownian diffusivity and the Stokes speed of the particle owing to gravity, affect parameters p and q.

Now using the definition for fractional wave variable v(x,t)=v(ξ), u(x,t)=u(ξ) and $\xi =K\frac{x^{\alpha }}{\alpha }+C\frac{t^{\alpha }}{\alpha }$ into Eqs. (19), (20) yields:

(20) $$Cu^{\prime }-K^{2}u^{\prime \prime }+2Kuu^{\prime }+pKuv^{\prime }+pKvu^{\prime }=0\text{,}$$

(21) $$Cv'-K^{2}v^{\prime \prime }+2Kvv'+qKuv^{\prime }+qKvu^{\prime }=0$$

### 4.1 The extension of the $\left(G^{\prime }/G\right)$-expansion method

The balancing value between $uu^{\prime }$ and $u^{\prime \prime }$ appeared in Eq. (20); $vv^{\prime }$ and $v^{\prime \prime }$ appeared in Eq. (21), yield $n_{1}=n_{2}=1$. Thus, the solution of Eqs. (20), (21) take the form:

(22) $$u\left(\xi \right)=a_{0}+a_{1}\left(d+1/\varphi \right)+b_{1}{\left(d+1/\varphi \right)}^{-1}$$

(23) $$v\left(\xi \right)=c_{0}+c_{1}\left(d+1/\varphi \right)+d_{1}{\left(d+1/\varphi \right)}^{-1}$$

where $a_{0},a_{1},b_{1},c_{0},c_{1},d_{1}$ and d are subjective constants to be calculated.

Swapping (22) and (23) together with (5), (6), (20), (21), the left-hand sides are changed into polynomial in ${\left(d+1/\varphi \right)}^{n},\left(n=0,1,2,\ldots \right)$ and ${\left(d+1/\varphi \right)}^{-n}$, (n=1,2,…). Collecting the cohorts of comparable power of these polynomials to zero, give two sets of equations (which are not presented her for simplicity) relating to $a_{0},a_{1},b_{1},c_{0},c_{1},d_{1},d,K$ and C. These equations are unravelled using the computation software Maple; we attain the subsequent three different sets of results.

**Set 1**:

$$a_{1}=K\mu \left(\frac{p-1}{pq-1}\right),b_{1}=0,c_{0}=\frac{a_{0}\left(q-1\right)}{p-1},C=-K\left(\frac{2Kd\mu p-2Kd\mu -K\lambda p+2a_{0}pq+K\lambda -2a_{0}}{p-1}\right)\text{,}$$

(24) $$d_{1}=0,c_{1}=K\mu \left(\frac{q-1}{pq-1}\right)\text{,}$$

where $a_{0},d,K,\lambda$ and μ are free parameters.

For Set 1, substituting the assessment amassed in (24) into solutions (22) and (23), together with (7), (8), (9), we obtain a series of wave results of (18), (19).

When $\rho =\lambda^{2}-4\mu >0$, we achieve the succeeding hyperbolic function solutions

$${u_{1}}_{1}\left(\xi \right)=a_{0}+K\mu \left(\frac{p-1}{pq-1}\right)\left\{d+\frac{2}{-\lambda +\sqrt{\rho }\;\left(\frac{\sinh\left(\xi \frac{\sqrt{\rho }}{2}\right)A_{1}+\cosh\left(\xi \frac{\sqrt{\rho }}{2}\right)A_{2}}{\cosh\left(\xi \frac{\sqrt{\rho }}{2}\right)\;A_{1}+\sinh\left(\xi \frac{\sqrt{\rho }}{2}\right)A_{2}}\right)}\right\}\text{,}$$

$${v_{1}}_{1}\left(\xi \right)=a_{0}\left(\frac{q-1}{p-1}\right)+K\mu \left(\frac{q-1}{pq-1}\right)\left\{d+\frac{2}{-\lambda +\sqrt{\rho }\;\left(\frac{\sinh\left(\xi \frac{\sqrt{\rho }}{2}\right)A_{1}+\cosh\left(\xi \frac{\sqrt{\rho }}{2}\right)A_{2}}{\cosh\left(\xi \frac{\sqrt{\rho }}{2}\right)\;A_{1}+\sinh\left(\xi \frac{\sqrt{\rho }}{2}\right)A_{2}}\right)}\right\}\text{.}$$

When $\rho =\lambda^{2}-4\mu <0$, we attain the resulting trigonometric solutions

$${u_{1}}_{2}\left(\xi \right)=a_{0}+K\mu \left(\frac{p-1}{pq-1}\right)\left\{d+\frac{2}{-\lambda +\sqrt{-\rho }\left(\frac{\sin\left(\xi \frac{\sqrt{-\rho }}{2}\right)A_{1}+\cos\left(\xi \frac{\sqrt{-\rho }}{2}\right)\;A_{2}}{\cos\left(\xi \frac{\sqrt{-\rho }}{2}\right)A_{1}+\sin\left(\xi \frac{\sqrt{-\rho }}{2}\right)A_{2}}\right)}\right\}\text{,}$$

$${v_{1}}_{2}\left(\xi \right)=a_{0}\left(\frac{q-1}{p-1}\right)+K\mu \left(\frac{q-1}{pq-1}\right)\left\{d+\frac{2}{-\lambda +\sqrt{-\rho }\left(\frac{\sin\left(\xi \frac{\sqrt{-\rho }}{2}\right)A_{1}+\cos\left(\xi \frac{\sqrt{-\rho }}{2}\right)\;A_{2}}{\cos\left(\xi \frac{\sqrt{-\rho }}{2}\right)A_{1}+\sin\left(\xi \frac{\sqrt{-\rho }}{2}\right)A_{2}}\right)}\right\}\text{.}$$

When $\rho =\lambda^{2}-4\mu =0$, we achieve the next rational solutions

$${u_{1}}_{3}\left(\xi \right)=a_{0}+K\mu \left(\frac{p-1}{pq-1}\right)\left(d+{\left(-\frac{\lambda }{2}+\frac{A_{2}}{A_{1}+A_{2}\xi }\right)}^{-1}\right)\text{,}$$

$${v_{1}}_{3}\left(\xi \right)=a_{0}\left(\frac{q-1}{p-1}\right)+K\mu \left(\frac{q-1}{pq-1}\right)\left(d+{\left(-\frac{\lambda }{2}+\frac{A_{2}}{A_{1}+A_{2}\xi }\right)}^{-1}\right)\text{,}$$

where $\xi =\frac{K}{\alpha }x^{\alpha }-\frac{K}{\alpha }\left(\frac{2Kd\mu p-2Kd\mu -K\lambda p+2a_{0}pq+K\lambda -2a_{0}}{p-1}\right)t^{\alpha }$.

**Set 2**:

$$a_{0}=c_{0}\left(\frac{p-1}{q-1}\right),b_{1}=-K\left(\frac{d^{2}\mu p-d^{2}\mu -d\lambda p+d\lambda +p-1}{pq-1}\right),d_{1}=-K\left(d^{2}\mu -d\lambda +1\right)\left(\frac{q-1}{pq-1}\right)\text{,}$$

(25) $$a_{1}=0,c_{1}=0,C=K\left(\frac{2Kd\mu q-2Kd\mu -K\lambda q-2c_{0}pq+K\lambda +2c_{0}}{q-1}\right)\text{,}$$

where $c_{0},d,K,\lambda$ and μ are random parameters.

For Set 2, substituting (25) into solutions (22) and (23), along with (7), (8), (9), we obtain a series of wave solutions to Eqs. (18), (19) respectively.

When $\rho =\lambda^{2}-4\mu >0$, we find the ensuing hyperbolic solutions

$${u_{2}}_{1}\left(\xi \right)=c_{0}\left(\frac{p-1}{q-1}\right)-K\left(\frac{\left(p-1\right)\left(d^{2}\mu -d\lambda +1\right)}{pq-1}\right)\{{d+\frac{2}{-\lambda +\sqrt{\rho }\;\left(\frac{\sinh\left(\xi \frac{\sqrt{\rho }}{2}\right)A_{1}+\cosh\left(\xi \frac{\sqrt{\rho }}{2}\right)A_{2}}{\cosh\left(\xi \frac{\sqrt{\rho }}{2}\right)A_{1}+\sinh\left(\xi \frac{\sqrt{\rho }}{2}\right)A_{2}}\right)}\}}^{-1}\text{,}$$

$${v_{2}}_{1}\left(\xi \right)=c_{0}-K\left(d^{2}\mu -d\lambda +1\right)\left(\frac{q-1}{pq-1}\right)\{{d+\frac{2}{-\lambda +\sqrt{\rho }\;\left(\frac{\sinh\left(\xi \frac{\sqrt{\rho }}{2}\right)A_{1}+\cosh\left(\xi \frac{\sqrt{\rho }}{2}\right)A_{2}}{\cosh\left(\xi \frac{\sqrt{\rho }}{2}\right)A_{1}+\sinh\left(\xi \frac{\sqrt{\rho }}{2}\right)A_{2}}\right)}\}}^{-1}\text{.}$$

When $\rho =\lambda^{2}-4\mu <0$, we derive the under mentioned trigonometric solutions

$${u_{2}}_{2}\left(\xi \right)=c_{0}\left(\frac{p-1}{q-1}\right)-K\left(\frac{\left(p-1\right)\left(d^{2}\mu -d\lambda +1\right)}{pq-1}\right)\{{d+\frac{2}{-\lambda +\sqrt{-\rho }\left(\frac{-\sin\left(\xi \frac{\sqrt{-\rho }}{2}\right)A_{1}+\cos\left(\xi \frac{\sqrt{-\rho }}{2}\right)A_{2}}{\cos\left(\xi \frac{\sqrt{-\rho }}{2}\right)A_{1}+\sin\left(\xi \frac{\sqrt{-\rho }}{2}\right)A_{2}}\right)}\}}^{-1}\text{,}$$

$${v_{2}}_{2}\left(\xi \right)=c_{0}-K\left(d^{2}\mu -d\lambda +1\right)\left(\frac{q-1}{pq-1}\right)\{{d+\frac{2}{-\lambda +\sqrt{-\rho }\left(\frac{{-A}_{1}\;\sin\left(\xi \frac{\sqrt{-\rho }}{2}\right){+A}_{2}\;\cos\left(\xi \frac{\sqrt{-\rho }}{2}\right)}{A_{1}\;\cos\left(\xi \frac{\sqrt{-\rho }}{2}\right)+A_{2}\;\sin\left(\xi \frac{\sqrt{-\rho }}{2}\right)}\right)}\}}^{-1}\text{.}$$

When $\rho =\lambda^{2}-4\mu =0$, we gain the rational solutions provided in the underneath

$${u_{2}}_{3}\left(\xi \right)=c_{0}\left(\frac{p-1}{q-1}\right)-K\left(\frac{d^{2}\mu p-d^{2}\mu -d\lambda p+d\lambda +p-1}{pq-1}\right){\left(d+{\left(-\frac{\lambda }{2}+\frac{A_{2}}{A_{1}+A_{2}\xi }\right)}^{-1}\right)}^{-1}\text{,}$$

$${v_{2}}_{3}\left(\xi \right)=c_{0}-K\left(d^{2}\mu -d\lambda +1\right)\left(\frac{q-1}{pq-1}\right){\left(d+{\left(-\frac{\lambda }{2}+\frac{A_{2}}{A_{1}+A_{2}\xi }\right)}^{-1}\right)}^{-1}\text{,}$$

where $\xi =\frac{K}{\alpha }x^{\alpha }+\frac{K}{\alpha }\left(\frac{2Kd\mu q-2Kd\mu -K\lambda q-2c_{0}pq+K\lambda +2c_{0}}{q-1}\right)t^{\alpha }$.

**Set 3**:

$$a_{1}=K\mu \left(\frac{p-1}{pq-1}\right),b_{1}=\frac{K}{4\mu }\left(\frac{\lambda^{2}p-\lambda^{2}-4\mu p+4\mu }{pq-1}\right),c_{0}=\frac{a_{0}\left(q-1\right)}{p-1},c_{1}=K\mu \left(\frac{q-1}{pq-1}\right),d=\frac{\lambda }{2\mu }\text{,}$$

(26) $$d_{1}=\frac{K}{4\mu }\left(\lambda^{2}-4\mu \right)\left(\frac{q-1}{pq-1}\right),C=-2Ka_{0}\left(\frac{pq-1}{p-1}\right)\text{,}$$

where $a_{0},K,\lambda$ and μ are free parameters.

For Set 3, substituting the values presented in (26) into solutions (22) and (23), together with (7), (8), (9), we obtain scores of closed-form solutions of (18), (19) respectively.

When $\rho =\lambda^{2}-4\mu >0$, we acquire the next hyperbolic function solutions

$${u_{3}}_{1}\left(\xi \right)=a_{0}+K\mu \left(\frac{p-1}{pq-1}\right)\left(\frac{\lambda }{2\mu }+Q_{1}\right)+\frac{K}{4\mu }\left(\frac{\lambda^{2}p-\lambda^{2}-4\mu p+4\mu }{pq-1}\right){\left(\frac{\lambda }{2\mu }+Q_{1}\right)}^{-1}$$

$${v_{3}}_{1}\left(\xi \right)=\frac{a_{0}\left(q-1\right)}{p-1}+K\mu \left(\frac{q-1}{pq-1}\right)\left(\frac{\lambda }{2\mu }+Q_{1}\right)+\frac{K}{4\mu }\left(\lambda^{2}-4\mu \right)\left(\frac{q-1}{pq-1}\right){\left(\frac{\lambda }{2\mu }+Q_{1}\right)}^{-1}$$

where $Q_{1}={\left\{-\frac{\lambda }{2}+\frac{\sqrt{\rho }}{2}\;\left(\frac{A_{1}\;\sinh\left(\xi \frac{\sqrt{\rho }}{2}\right){+A}_{2}\;\cosh\left(\xi \frac{\sqrt{\rho }}{2}\right)}{A_{1}\;\cosh\left(\xi \frac{\sqrt{\rho }}{2}\right)+A_{2}\;\sinh\left(\xi \frac{\sqrt{\rho }}{2}\right)}\right)\right\}}^{-1}$.

When $\rho =\lambda^{2}-4\mu <0$, the resulting trigonometric solutions are established

$${u_{3}}_{2}\left(\xi \right)=a_{0}+K\mu \left(\frac{p-1}{pq-1}\right)\left(\frac{\lambda }{2\mu }+Q_{2}\right)+\frac{K}{4\mu }\left(\frac{\lambda^{2}p-\lambda^{2}-4\mu p+4\mu }{pq-1}\right){\left(\frac{\lambda }{2\mu }+Q_{2}\right)}^{-1}\text{,}$$

$${v_{3}}_{2}\left(\xi \right)=\frac{a_{0}\left(q-1\right)}{p-1}+K\mu \left(\frac{q-1}{pq-1}\right)\left(\frac{\lambda }{2\mu }+Q_{2}\right)+\frac{K}{4\mu }\left(\lambda^{2}-4\mu \right)\left(\frac{q-1}{pq-1}\right){\left(\frac{\lambda }{2\mu }+Q_{2}\right)}^{-1}\text{,}$$

where $Q_{2}={\left\{-\frac{\lambda }{2}+\frac{\sqrt{-\rho }}{2}\;\left(\frac{{-A}_{1}\;\sin\left(\xi \frac{\sqrt{-\rho }}{2}\right){+A}_{2}\;\cos\left(\xi \frac{\sqrt{-\rho }}{2}\right)}{A_{1}\;\cos\left(\xi \frac{\sqrt{-\rho }}{2}\right)+A_{2}\;\sin\left(\xi \frac{\sqrt{-\rho }}{2}\right)}\right)\right\}}^{-1}$.

When $\rho =\lambda^{2}-4\mu =0$, the following rational solutions are derived

$${u_{3}}_{3}\left(\xi \right)=a_{0}+K\mu \left(\frac{p-1}{pq-1}\right)\left(\frac{\lambda }{2\mu }+{\left(-\frac{\lambda }{2}+\frac{A_{2}}{A_{1}+A_{2}\xi }\right)}^{-1}\right)\text{,}$$

$${v_{3}}_{3}\left(\xi \right)=a_{0}\left(\frac{q-1}{p-1}\right)+K\mu \left(\frac{q-1}{pq-1}\right)\left(\frac{\lambda }{2\mu }+{\left(-\frac{\lambda }{2}+\frac{A_{2}}{A_{1}+A_{2}\xi }\right)}^{-1}\right)\text{,}$$

where $\xi =\frac{K}{\alpha }x^{\alpha }-\frac{2K}{\alpha }a_{0}\left(\frac{pq-1}{p-1}\right)t^{\alpha }$.

### 4.2 The extension of the generalized $\left(G^{\prime }/G\right)$-expansion method

Likewise, by means homogeneous balance between $uu^{\prime }$ and $u^{\prime \prime }$ in Eq. (20); $vv^{\prime }$ and $v^{\prime \prime }$ in Eq. (21), yield $n_{1}=n_{2}=1$. Therefore, the solution's shapes are similar to those of (22), (23), and for simplicity, they are not repeated here.

Therefore, stand in for solutions (22) and (23) along with (11), (12) into Eqs. (20), (21), the left-hand sides are adapted into polynomial in ${\left(d+1/\varphi \right)}^{n},\left(n=0,1,2,\ldots \right)$ and ${\left(d+1/\varphi \right)}^{-n},\left(n=1,2,\ldots \right)$. Collecting the coefficients of like power of the polynomial to zero, yield two sets of algebraic equations (which are untaken here for easiness) for $a_{0},a_{1},b_{1},c_{0},c_{1},d_{1},d,K$ and C. We resolve the algebraic equations with the help of Maple software package, we obtain four different sets of solutions as follows:

**Set 1**:

(27) $$a_{1}=-\frac{EK}{A}\frac{p-1}{pq-1},b_{1}=d_{1}=0,c_{0}=\frac{a_{0}\left(q-1\right)}{p-1},c_{1}=-\frac{EK}{A}\frac{q-1}{pq-1},C=-\frac{K}{A}\frac{2Aa_{0}\left(pq-1\right)+k\left(p-1\right)\left(B-2Ed\right)}{p-1}\text{,}$$

where K, $a_{0}$, A, B, d and E are random constants.

Embedding the estimations of the parameters assembled in (27) into solutions (22) and (23), together with Eqs. (13), (14), (15), (16), (17), we attain the subsequent series of wave solutions to Eqs. (18), (19) respectively.

If B≠0, $\Omega =B^{2}+4E\left(A-D\right)>0$, we obtain hyperbolic function solutions as:

$${u_{4}}_{1}\left(\xi \right)=a_{0}-\frac{EK}{A}\left(\frac{p-1}{pq-1}\right)\;\left\{d+\frac{2A-2D}{B+\sqrt{\Omega }\left(\frac{\sinh\left(\xi \frac{\sqrt{\Omega }}{2A}\right)A_{1}+\cosh\left(\xi \frac{\sqrt{\Omega }}{2A}\right)A_{2}}{\cosh\left(\xi \frac{\sqrt{\Omega }}{2A}\right)A_{1}+\sinh\left(\xi \frac{\sqrt{\Omega }}{2A}\right)A_{2}}\right)}\right\}\text{,}$$

$${v_{4}}_{1}\left(\xi \right)=a_{0}\left(\frac{q-1}{p-1}\right)-\frac{EK}{A}\left(\frac{q-1}{pq-1}\right)\left\{d+\frac{2A-2D}{B+\sqrt{\Omega }\left(\frac{\sinh\left(\xi \frac{\sqrt{\Omega }}{2A}\right)A_{1}+\cosh\left(\xi \frac{\sqrt{\Omega }}{2A}\right)A_{2}}{\cosh\left(\xi \frac{\sqrt{\Omega }}{2A}\right)A_{1}+\sinh\left(\xi \frac{\sqrt{\Omega }}{2A}\right)A_{2}}\right)}\right\}\text{.}$$

If B≠0, $\Omega =B^{2}+4E\left(A-D\right)<0$, we obtain trigonometric function solutions as:

$${u_{4}}_{2}\left(\xi \right)=a_{0}-\frac{EK}{A}\left(\frac{p-1}{pq-1}\right)\;\left\{d+\frac{2A-2D}{B+\sqrt{-\Omega }\left(\frac{{-A}_{1}\;\cos\frac{\sqrt{-\Omega }}{2A}\;\xi +A_{2}\;\sin\frac{\sqrt{-\Omega }}{2A}\;\xi }{A_{1}\;\sin\frac{\sqrt{-\Omega }}{2A}\;\xi +A_{2}\;\cos\frac{\sqrt{-\Omega }}{2A}\;\xi }\right)}\right\}\text{,}$$

$${v_{4}}_{2}\left(\xi \right)=a_{0}\left(\frac{q-1}{p-1}\right)-\frac{EK}{A}\left(\frac{q-1}{pq-1}\right)\left\{d+\frac{2A-2D}{B+\sqrt{-\Omega }\left(\frac{{-A}_{1}\;\cos\frac{\sqrt{-\Omega }}{2A}\;\xi +A_{2}\;\sin\frac{\sqrt{-\Omega }}{2A}\;\xi }{A_{1}\;\sin\frac{\sqrt{-\Omega }}{2A}\;\xi +A_{2}\;\cos\frac{\sqrt{-\Omega }}{2A}\;\xi }\right)}\right\}\text{.}$$

If B≠0, $\Omega =B^{2}+4E\left(A-D\right)=0$, we obtain rational form solutions as:

$${u_{4}}_{3}\left(\xi \right)=a_{0}-\frac{EK}{A}\left(\frac{p-1}{pq-1}\right)\left(d+{\left(\frac{B}{2A-2D}+\frac{A_{2}}{A_{1}+A_{2}\xi }\right)}^{-1}\right)\text{,}$$

$${v_{4}}_{3}\left(\xi \right)=a_{0}\left(\frac{q-1}{p-1}\right)-\frac{EK}{A}\left(\frac{q-1}{pq-1}\right)\left(d+{\left(\frac{B}{2A-2D}+\frac{A_{2}}{A_{1}+A_{2}\xi }\right)}^{-1}\right)\text{.}$$

If B=0, Δ=E(A−D)>0, we obtain hyperbolic function solutions as:

$${u_{4}}_{4}\left(\xi \right)=a_{0}-\frac{EK}{A}\left(\frac{p-1}{pq-1}\right)\left\{d+\frac{A-D}{\sqrt{\Delta }}\left(\frac{\cosh\left(\xi \frac{\sqrt{\Delta }}{A}\right)A_{1}+\sinh\left(\xi \frac{\sqrt{\Delta }}{A}\right)A_{2}}{\sinh\left(\xi \frac{\sqrt{\Delta }}{A}\right)A_{1}+\cosh\left(\xi \frac{\sqrt{\Delta }}{A}\right)A_{2}}\right)\right\}\text{,}$$

$${v_{4}}_{4}\left(\xi \right)=a_{0}\left(\frac{q-1}{p-1}\right)-\frac{EK}{A}\left(\frac{q-1}{pq-1}\right)\left\{d+\frac{A-D}{\sqrt{\Delta }}\left(\frac{A_{1}\;\cosh\frac{\sqrt{\Delta }}{A}\;\xi +A_{2}\;\sinh\frac{\sqrt{\Delta }}{A}\;\xi }{A_{1}\;\sinh\frac{\sqrt{\Delta }}{A}\;\xi +A_{2}\;\cosh\frac{\sqrt{\Delta }}{A}\;\xi }\right)\right\}\text{.}$$

If B=0, Δ=E(A−D)<0, we obtain trigonometric function solutions as:

$${u_{4}}_{5}\left(\xi \right)=a_{0}-\frac{EK}{A}\left(\frac{p-1}{pq-1}\right)\left\{d+\frac{A-D}{\sqrt{-\Delta }}\left(\frac{\sin\left(\frac{\sqrt{-\Delta }}{A}\;\xi \right)\;A_{1}+\cos\left(\frac{\sqrt{-\Delta }}{A}\;\xi \right)\;A_{2}}{-\cos\left(\frac{\sqrt{-\Delta }}{A}\;\xi \right)\;A_{1}+A_{2}\;\sin\frac{\sqrt{-\Delta }}{A}\;\xi }\right)\right\}\text{,}$$

$${v_{4}}_{5}\left(\xi \right)=a_{0}\left(\frac{q-1}{p-1}\right)-\frac{EK}{A}\left(\frac{q-1}{pq-1}\right)\left\{d+\frac{A-D}{\sqrt{-\Delta }}\left(\frac{A_{1}\;\sin\frac{\sqrt{-\Delta }}{A}\;\xi +A_{2}\;\cos\frac{\sqrt{-\Delta }}{A}\;\xi }{-A_{1}\;\cos\frac{\sqrt{-\Delta }}{A}\;\xi +{\sin\left(\frac{\sqrt{-\Delta }}{A}\;\xi \right)\;A}_{2}}\right)\right\}\text{,}$$

where $\xi =\frac{K}{\alpha }x^{\alpha }-\frac{K}{\alpha A}\left(\frac{2Aa_{0}pq-2EKdp+BKp+2EKd-2Aa_{0}-BK}{p-1}\right)t^{\alpha }$.

**Set 2**:

$$b_{1}=-\frac{K}{A}\frac{-Epd^{2}+Bpd+Ed^{2}-Bd+p\left(A-D\right)-A+D}{pq-1},d_{1}=-\frac{K}{A}\frac{-Eqd^{2}+Bqd+Ed^{2}-Bd+q\left(A-D\right)-A+D}{pq-1}\text{,}$$

(28) $$c_{0}=\frac{a_{0}\left(q-1\right)}{p-1},c_{1}=0,a_{1}=0,C=-\frac{K}{A}\frac{2Aa_{0}pq+2EKdp-BKp-2EKd-2Aa_{0}+BK}{p-1}\text{,}$$

where $a_{0}$, K, d, E, D, A, and B are random parameters.

Swapping the values of the parameters from (28), (22), (23), along with (13), (14), (15), (16), (17), we acquire the subsequent solutions to Eqs. (18), (19) respectively.

If B≠0, $\Omega =B^{2}+4E\left(A-D\right)>0$, we obtain hyperbolic function solutions as:

$${u_{5}}_{1}\left(\xi \right)=a_{0}-\frac{K}{A}\left(\frac{\left(p-1\right)\left(Bd-Ed^{2}+A-D\right)}{pq-1}\right)\{{d+\frac{2A-2D}{B+\sqrt{\Omega }\left(\frac{\sinh\left(\xi \frac{\sqrt{\Omega }}{2A}\right)A_{1}+\cosh\left(\xi \frac{\sqrt{\Omega }}{2A}\right)A_{2}}{\cosh\left(\xi \frac{\sqrt{\Omega }}{2A}\right)A_{1}+\sinh\left(\xi \frac{\sqrt{\Omega }}{2A}\right)A_{2}}\right)}\}}^{-1}\text{,}$$

$${v_{5}}_{1}\left(\xi \right)=a_{0}\left(\frac{q-1}{p-1}\right)-\frac{K}{A}\left(\frac{\left(q-1\right)\left(Bd-Ed^{2}+A-D\right)}{pq-1}\right)\{{d+\frac{2A-2D}{B+\sqrt{\Omega }\left(\frac{\sinh\left(\xi \frac{\sqrt{\Omega }}{2A}\right)A_{1}+\cosh\left(\xi \frac{\sqrt{\Omega }}{2A}\right)A_{2}}{\cosh\left(\xi \frac{\sqrt{\Omega }}{2A}\right)A_{1}+\sinh\left(\xi \frac{\sqrt{\Omega }}{2A}\right)A_{2}}\right)}\}}^{-1}\text{.}$$

If B≠0, $\Omega =B^{2}+4E\left(A-D\right)<0$, we obtain trigonometric function solutions as:

$${u_{5}}_{2}\left(\xi \right)=a_{0}-\frac{K}{A}\left(\frac{\left(p-1\right)\left(Bd-Ed^{2}+A-D\right)}{pq-1}\right){\left\{d+\frac{2A-2D}{B+\sqrt{-\Omega }\left(\frac{{-A}_{1}\;\sin\left(\xi \frac{\sqrt{-\Omega }}{2A}\right)+A_{2}\;\cos\left(\xi \frac{\sqrt{-\Omega }}{2A}\right)}{\cos\left(\xi \frac{\sqrt{-\Omega }}{2A}\right)\;A_{1}+\sin\left(\xi \frac{\sqrt{-\Omega }}{2A}\right)A_{2}}\right)}\right\}}^{-1}\text{,}$$

$${v_{5}}_{2}\left(\xi \right)=a_{0}\frac{q-1}{p-1}-\frac{K}{A}\left(\frac{\left(q-1\right)\left(Bd-Ed^{2}+A-D\right)}{pq-1}\right){\left\{d+\frac{2A-2D}{B+\sqrt{-\Omega }\left(\frac{{-A}_{1}\;\sin\left(\xi \frac{\sqrt{-\Omega }}{2A}\right)+A_{2}\;\cos\left(\xi \frac{\sqrt{-\Omega }}{2A}\right)}{\cos\left(\xi \frac{\sqrt{-\Omega }}{2A}\right)\;A_{1}+\sin\left(\xi \frac{\sqrt{-\Omega }}{2A}\right)A_{2}}\right)}\right\}}^{-1}\text{.}$$

If B≠0, $\Omega =B^{2}+4E\left(A-D\right)=0$, we obtain rational form solutions as:

$${u_{5}}_{3}\left(\xi \right)=a_{0}-\frac{K}{A}\left(\frac{\left(p-1\right)\left(Bd-Ed^{2}+A-D\right)}{pq-1}\right)\;{\left\{d+{\left(\frac{B}{2A-2D}+\frac{A_{2}}{A_{1}+A_{2}\xi }\right)}^{-1}\right\}}^{-1}\text{,}$$

$${v_{5}}_{3}\left(\xi \right)=a_{0}\left(\frac{q-1}{p-1}\right)-\frac{K}{A}\left(\frac{\left(q-1\right)\left(Bd-Ed^{2}+A-D\right)}{pq-1}\right){\left\{d+{\left(\frac{B}{2A-2D}+\frac{A_{2}}{A_{1}+A_{2}\xi }\right)}^{-1}\right\}}^{-1}\text{.}$$

If B=0,Δ=E(A−D)>0, we obtain hyperbolic function solutions as:

$${u_{5}}_{4}\left(\xi \right)=a_{0}-\frac{K}{A}\left(\frac{\left(p-1\right)\left(Bd-Ed^{2}+A-D\right)}{pq-1}\right)\;{\left\{d+\frac{A-D}{\sqrt{\Delta }}\left(\frac{A_{1}\;\cosh\frac{\sqrt{\Delta }}{A}\;\xi +A_{2}\;\sinh\frac{\sqrt{\Delta }}{A}\;\xi }{A_{1}\;\sinh\frac{\sqrt{\Delta }}{A}\;\xi +A_{2}\;\cosh\frac{\sqrt{\Delta }}{A}\;\xi }\right)\right\}}^{-1}\text{,}$$

$${v_{5}}_{4}\left(\xi \right)=a_{0}\left(\frac{q-1}{p-1}\right)-\frac{K}{A}\left(\frac{\left(q-1\right)\left(A-D-Ed^{2}\right)}{pq-1}\right){\left(d+\frac{A-D}{\sqrt{\Delta }}\left(\frac{A_{1}\;\cosh\frac{\sqrt{\Delta }}{A}\;\xi +A_{2}\;\sinh\frac{\sqrt{\Delta }}{A}\;\xi }{A_{1}\;\sinh\frac{\sqrt{\Delta }}{A}\;\xi +A_{2}\;\cosh\frac{\sqrt{\Delta }}{A}\;\xi }\right)\right)}^{-1}\text{.}$$

If B=0, Δ=E(A−D)<0, we obtain trigonometric function solutions as:

$${u_{5}}_{5}\left(\xi \right)=a_{0}-\frac{K}{A}\left(\frac{\left(q-1\right)\left(A-D-Ed^{2}\right)}{pq-1}\right){\left\{d+\frac{A-D}{\sqrt{-\Delta }}\left(\frac{A_{1}\;\cos\frac{\sqrt{-\Delta }}{A}\;\xi +A_{2}\;\sin\frac{\sqrt{-\Delta }}{A}\;\xi }{-A_{1}\;\sin\frac{\sqrt{-\Delta }}{A}\;\xi +A_{2}\;\cos\frac{\sqrt{-\Delta }}{A}\;\xi }\right)\right\}}^{-1}\text{,}$$

$${v_{5}}_{5}\left(\xi \right)=a_{0}\left(\frac{q-1}{p-1}\right)-\frac{K}{A}\left(\frac{\left(q-1\right)\left(A-D-Ed^{2}\right)}{pq-1}\right){\left(d+\frac{A-D}{\sqrt{-\Delta }}\left(\frac{A_{1}\;\cos\frac{\sqrt{-\Delta }}{A}\;\xi +A_{2}\;\sin\frac{\sqrt{-\Delta }}{A}\;\xi }{-A_{1}\;\sin\frac{\sqrt{-\Delta }}{A}\;\xi +A_{2}\;\cos\frac{\sqrt{-\Delta }}{A}\;\xi }\right)\right)}^{-1}\text{,}$$

where $\xi =\frac{K}{\alpha }x^{\alpha }-\frac{K}{\alpha A}\left(\frac{2Aa_{0}pq+2EKdp-BKp-2EKd-2Aa_{0}+BK}{p-1}\right)t^{\alpha }$.

**Set 3**:

$$a_{1}=-\frac{EK}{A}\frac{p-1}{pq-1},b_{1}=-\frac{K}{4AE}\frac{B^{2}p+4Ep\left(A-D\right)-B^{2}-4E\left(A-D\right)}{pq-1},c_{0}=\frac{a_{0}\left(q-1\right)}{p-1},d=\frac{B}{2E}\text{,}$$

(29) $$c_{1}=-\frac{EK}{A}\frac{q-1}{pq-1},d_{1}=-\frac{K}{4AE}\frac{B^{2}q+4Eq\left(A-D\right)-B^{2}-4E\left(A-D\right)}{pq-1},C=-\frac{2a_{0}K\left(pq-1\right)}{p-1}\text{,}$$

where $a_{0},A,B,D,E$ and K are free parameters.

For Set 3, substituting Eq. (29) into Eqs. (22), (23), together with Eqs. (13), (14), (15), (16), (17), we obtain a series of exact wave solutions to Eqs. (18), (19) respectively.

If $B\neq 0,\Omega =B^{2}+4E\left(A-D\right)>0$, we obtain hyperbolic function solutions as:

$${u_{6}}_{1}\left(\xi \right)=a_{0}-\frac{EK}{A}\left(\frac{p-1}{pq-1}\right)\left(\frac{B}{2E}+P_{1}\right)-\frac{K}{4AE}\left(\frac{\left(p-1\right)\left(4E\left(A-D\right)+B^{2}\right)}{pq-1}\right){\left(\frac{B}{2E}+P_{1}\right)}^{-1}\text{,}$$

$${v_{6}}_{1}\left(\xi \right)=a_{0}\left(\frac{q-1}{p-1}\right)-\frac{EK}{A}\left(\frac{q-1}{pq-1}\right)\left(\frac{B}{2E}+P_{1}\right)-\frac{K}{4AE}\left(\frac{\left(q-1\right)\left(4E\left(A-D\right)+B^{2}\right)}{pq-1}\right){\left(\frac{B}{2E}+P_{1}\right)}^{-1}\text{,}$$

where $P_{1}=2{\left(\frac{B}{A-D}+\frac{\sqrt{\Omega }}{A-D}\left(\frac{\sinh\left(\frac{\sqrt{\Omega }}{2A}\;\xi \right)\;A_{1}+\cosh\left(\frac{\sqrt{\Omega }}{2A}\;\xi \right)\;A_{2}}{\cosh\left(\frac{\sqrt{\Omega }}{2A}\;\xi \right)\;A_{1}+\sinh\left(\frac{\sqrt{\Omega }}{2A}\;\xi \right)\;A_{2}}\right)\right)}^{-1}$.

If B≠0, $\Omega =B^{2}+4E\left(A-D\right)<0$, we obtain trigonometric function solutions as:

$${u_{6}}_{2}\left(\xi \right)=a_{0}-\frac{EK}{A}\left(\frac{p-1}{pq-1}\right)\left(\frac{B}{2E}+P_{2}\right)-\frac{K}{4AE}\left(\frac{\left(p-1\right)\left(4E\left(A-D\right)+B^{2}\right)}{pq-1}\right){\left(\frac{B}{2E}+P_{2}\right)}^{-1}\text{,}$$

$${v_{6}}_{2}\left(\xi \right)=a_{0}\left(\frac{q-1}{p-1}\right)-\frac{EK}{A}\left(\frac{q-1}{pq-1}\right)\left(\frac{B}{2E}+P_{2}\right)-\frac{K}{4AE}\left(\frac{\left(q-1\right)\left(4E\left(A-D\right)+B^{2}\right)}{pq-1}\right){\left(\frac{B}{2E}+P_{2}\right)}^{-1}\text{,}$$

where $P_{2}=2{\left(\frac{B}{A-D}+\frac{\sqrt{-\Omega }}{A-D}\left(\frac{{-\sin\left(\frac{\sqrt{-\Omega }}{2A}\;\xi \right)\;A}_{1}+{\cos\left(\frac{\sqrt{-\Omega }}{2A}\;\xi \right)\;A}_{2}}{\cos\left(\frac{\sqrt{-\Omega }}{2A}\right)\;\xi \;A_{1}+\sin\left(\frac{\sqrt{-\Omega }}{2A}\;\xi \right)\;A_{2}}\right)\right)}^{-1}$.

If B≠0, $\Omega =B^{2}+4E\left(A-D\right)=0$, we obtain rational form solutions as:

$${u_{6}}_{3}\left(\xi \right)=a_{0}-\frac{EK}{A}\left(\frac{p-1}{pq-1}\right)\left(\frac{B}{2E}+{\left(\frac{B}{2A-2D}+\frac{A_{2}}{A_{1}+A_{2}\xi }\right)}^{-1}\right)\text{,}$$

$${v_{6}}_{3}\left(\xi \right)=a_{0}\left(\frac{q-1}{p-1}\right)-\frac{EK}{A}\left(\frac{q-1}{pq-1}\right)\left(\frac{B}{2E}+{\left(\frac{B}{2A-2D}+\frac{A_{2}}{A_{1}+A_{2}\xi }\right)}^{-1}\right)\text{.}$$

If B=0, Δ=E(A−D)>0, we obtain hyperbolic function solutions as:

$${u_{6}}_{4}\left(\xi \right)=a_{0}-\frac{EK}{A}\left(\frac{p-1}{pq-1}\right)\left(P_{3}\right)-\frac{K}{A}\left(A-D\right)\left(\frac{p-1}{pq-1}\right){\left(P_{3}\right)}^{-1}\text{,}$$

$${v_{6}}_{4}\left(\xi \right)=a_{0}\left(\frac{q-1}{p-1}\right)-\frac{EK}{A}\left(\frac{q-1}{pq-1}\right)\left(P_{3}\right)-\frac{K}{A}\left(A-D\right)\left(\frac{q-1}{pq-1}\right){\left(P_{3}\right)}^{-1}\text{,}$$

where $P_{3}=\frac{A-D}{\sqrt{\Delta }}\left(\frac{\sinh\left(\frac{\sqrt{\Delta }}{2A}\;\xi \right)\;A_{1}+\cosh\left(\frac{\sqrt{\Delta }}{2A}\;\xi \right)\;A_{2}}{\cosh\left(\frac{\sqrt{\Delta }}{2A}\;\xi \right)\;A_{1}+\sinh\left(\frac{\sqrt{\Delta }}{2A}\;\xi \right)\;A_{2}}\right)$.

If B=0,Δ=E(A−D)<0, we obtain trigonometric function solutions as:

$${u_{6}}_{5}\left(\xi \right)=a_{0}-\frac{EK}{A}\left(\frac{p-1}{pq-1}\right)\left(P_{4}\right)-\frac{K}{A}\left(A-D\right)\left(\frac{p-1}{pq-1}\right){\left(P_{4}\right)}^{-1}\text{,}$$

$${v_{6}}_{5}\left(\xi \right)=a_{0}\left(\frac{q-1}{p-1}\right)-\frac{EK}{A}\left(\frac{q-1}{pq-1}\right)\left(P_{4}\right)-\frac{K}{A}\left(A-D\right)\left(\frac{q-1}{pq-1}\right){\left(P_{4}\right)}^{-1}\text{,}$$

where $P_{4}=\frac{A-D}{\sqrt{-\Delta }}\left(\frac{A_{1}\;\cos\frac{\sqrt{-\Delta }}{A}\;\xi +A_{2}\;\sin\frac{\sqrt{-\Delta }}{A}\;\xi }{-A_{1}\;\sin\frac{\sqrt{-\Delta }}{A}\;\xi +A_{2}\;\cos\frac{\sqrt{-\Delta }}{A}\;\xi }\right)$ and $\xi =\frac{K}{\alpha }x^{\alpha }-\frac{2Ka_{0}}{\alpha }\left(\frac{pq-1}{p-1}\right)t^{\alpha }$.

**Set 4**:

$$a_{0}=0,a_{1}=0,b_{1}=-\frac{K}{A}\frac{\left(p-1\right)\left(Bd-Ed^{2}+A-D\right)}{pq-1},c_{0}=0,c_{1}=0\text{,}$$

(30) $$d_{1}=-\frac{K}{A}\frac{\left(q-1\right)\left(Bd-Ed^{2}+A-D\right)}{pq-1},C=-\frac{K^{2}}{A}\left(2Ed-B\right)\text{,}$$

where d,A,B,D,E and K are free parameters.

For Set 4, substituting (30) into solutions (22) and (23), along with (13), (14), (15), (16), (17), we obtain a series of wave solutions to Eqs. (18), (19) respectively.

If B≠0, $\Omega =B^{2}+4E\left(A-D\right)>0$, we obtain hyperbolic function solutions as:

$${u_{7}}_{1}\left(\xi \right)=-\frac{K}{A}\left(\frac{\left(p-1\right)\left(Bd-Ed^{2}+A-D\right)}{pq-1}\right){\left\{d+\frac{2A-2D}{B+\sqrt{\Omega }\left(\frac{A_{1}\;\sinh\frac{\sqrt{\Omega }}{2A}\;\xi +A_{2}\;\cosh\frac{\sqrt{\Omega }}{2A}\;\xi }{A_{1}\;\cosh\frac{\sqrt{\Omega }}{2A}\;\xi +A_{2}\;\sinh\frac{\sqrt{\Omega }}{2A}\;\xi }\right)}\right\}}^{-1}\text{,}$$

$${v_{7}}_{1}\left(\xi \right)=-\frac{K}{A}\frac{\left(q-1\right)\left(Bd-Ed^{2}+A-D\right)}{pq-1}{\left\{d+\frac{2A-2D}{B+\sqrt{\Omega }\left(\frac{\sinh\left(\xi \frac{\sqrt{\Omega }}{2A}\right)A_{1}+\cosh\left(\xi \frac{\sqrt{\Omega }}{2A}\right)A_{2}}{\cosh\left(\xi \frac{\sqrt{\Omega }}{2A}\right)A_{1}+\sinh\left(\xi \frac{\sqrt{\Omega }}{2A}\right)A_{2}}\right)}\right\}}^{-1}\text{.}$$

If $B\neq 0,\Omega =B^{2}+4E\left(A-D\right)<0$, we obtain trigonometric function solutions as:

$${u_{7}}_{2}\left(\xi \right)=-\frac{K}{A}\left(\frac{\left(p-1\right)\left(Bd-Ed^{2}+A-D\right)}{pq-1}\right){\left\{d+\frac{2A-2D}{B+\sqrt{-\Omega }\left(\frac{-\sin\left(\xi \frac{\sqrt{-\Omega }}{2A}\right)A_{1}+\cos\left(\xi \frac{\sqrt{-\Omega }}{2A}\right)A_{2}}{\cos\left(\xi \frac{\sqrt{-\Omega }}{2A}\right)A_{1}+\sin\left(\frac{\sqrt{-\Omega }}{2A}\;\xi \right)\;A_{2}}\right)}\right\}}^{-1}\text{,}$$

$${v_{7}}_{2}\left(\xi \right)=-\frac{K}{A}\frac{\left(q-1\right)\left(Bd-Ed^{2}+A-D\right)}{pq-1}{\left\{d+\frac{2A-2D}{B+\sqrt{-\Omega }\left(\frac{{-A}_{1}\;\sin\frac{\sqrt{-\Omega }}{2A}\;\xi +A_{2}\;\cos\frac{\sqrt{-\Omega }}{2A}\;\xi }{A_{1}\;\cos\frac{\sqrt{-\Omega }}{2A}\;\xi +A_{2}\;\sin\frac{\sqrt{-\Omega }}{2A}\;\xi }\right)}\right\}}^{-1}\text{.}$$

If $B\neq 0,\Omega =B^{2}+4E\left(A-D\right)=0$, we obtain rational form solutions as:

$${u_{7}}_{3}\left(\xi \right)=-\frac{K}{A}\left(\frac{\left(p-1\right)\left(Bd-Ed^{2}+A-D\right)}{pq-1}\right){\left\{d+{\left(\frac{B}{2A-2D}+\frac{A_{2}}{A_{1}+A_{2}\xi }\right)}^{-1}\right\}}^{-1}\text{,}$$

$${v_{7}}_{3}\left(\xi \right)=-\frac{K}{A}\left(\frac{\left(q-1\right)\left(Bd-Ed^{2}+A-D\right)}{pq-1}\right){\left\{d+{\left(\frac{B}{2A-2D}+\frac{A_{2}}{A_{1}+A_{2}\xi }\right)}^{-1}\right\}}^{-1}\text{.}$$

If B=0,Δ=E(A−D)>0, we obtain hyperbolic function solutions as:

$${u_{7}}_{4}\left(\xi \right)=-\frac{K}{A}\left(\frac{\left(p-1\right)\left(Bd-Ed^{2}+A-D\right)}{pq-1}\right){\left\{d+\frac{A-D}{\sqrt{\Delta }}\left(\frac{A_{1}\;\cosh\frac{\sqrt{\Delta }}{A}\;\xi +A_{2}\;\sinh\frac{\sqrt{\Delta }}{A}\;\xi }{A_{1}\;\sinh\frac{\sqrt{\Delta }}{A}\;\xi +A_{2}\;\cosh\frac{\sqrt{\Delta }}{A}\;\xi }\right)\right\}}^{-1}\text{,}$$

$${v_{7}}_{4}\left(\xi \right)=-\frac{K}{A}\left(\frac{\left(q-1\right)\left(Bd-Ed^{2}+A-D\right)}{pq-1}\right){\left\{d+\frac{A-D}{\sqrt{\Delta }}\left(\frac{\cosh\left(\xi \frac{\sqrt{\Delta }}{A}\right)A_{1}+\sinh\left(\xi \frac{\sqrt{\Delta }}{A}\right)\;A_{2}}{\sinh\left(\xi \frac{\sqrt{\Delta }}{A}\right)A_{1}+\cosh\left(\xi \frac{\sqrt{\Delta }}{A}\right)A_{2}}\right)\right\}}^{-1}\text{.}$$

If B=0,Δ=E(A−D)<0, we obtain trigonometric function solutions as:

$${u_{7}}_{5}\left(\xi \right)=-\frac{K}{A}\left(\frac{\left(p-1\right)\left(Bd-Ed^{2}+A-D\right)}{pq-1}\right){\left\{d+\frac{A-D}{\sqrt{-\Delta }}\left(\frac{A_{1}\;\cos\frac{\sqrt{-\Delta }}{A}\;\xi +A_{2}\;\sin\frac{\sqrt{-\Delta }}{A}\;\xi }{-A_{1}\;\sin\frac{\sqrt{-\Delta }}{A}\;\xi +A_{2}\;\cos\frac{\sqrt{-\Delta }}{A}\;\xi }\right)\right\}}^{-1}\text{,}$$

$${v_{7}}_{5}\left(\xi \right)=-\frac{K}{A}\left(\frac{\left(q-1\right)\left(Bd-Ed^{2}+A-D\right)}{pq-1}\right){\left\{d+\frac{A-D}{\sqrt{-\Delta }}\left(\frac{\cos\left(\xi \frac{\sqrt{-\Delta }}{A}\right)A_{1}+\sin\left(\xi \frac{\sqrt{-\Delta }}{A}\right)A_{2}}{-\sin\left(\xi \frac{\sqrt{-\Delta }}{A}\right)A_{1}+\cos\left(\xi \frac{\sqrt{-\Delta }}{A}\right)A_{2}}\right)\right\}}^{-1}\text{,}$$

where $\xi =\frac{K}{\alpha }x^{\alpha }-\frac{K^{2}}{\alpha A}\left(2Ed-B\right)t^{\alpha }$.

## 5 The numerical simulations

**Fig. 1:**
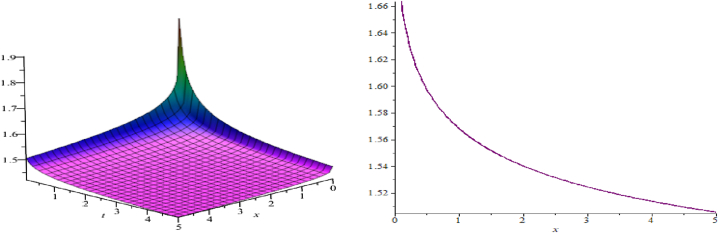
A soliton, modulus plot of solution ${u_{1}}_{3}$.

**Fig. 2:**
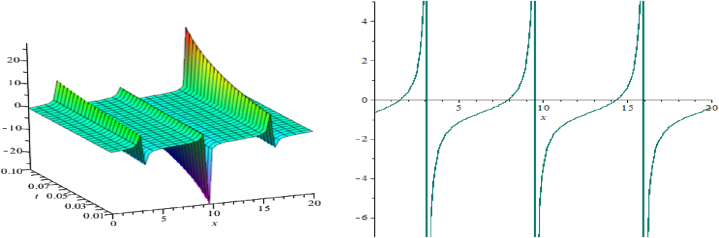
Singular periodic soliton, modulus plot of solution ${u_{2}}_{2}$.

**Fig. 3:**
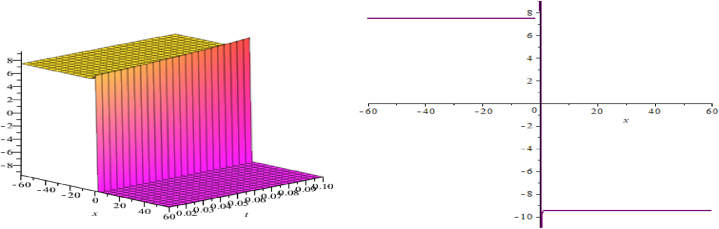
Kink type soliton, modulus plot of solution ${u_{3}}_{1}$.

**Fig. 4:**
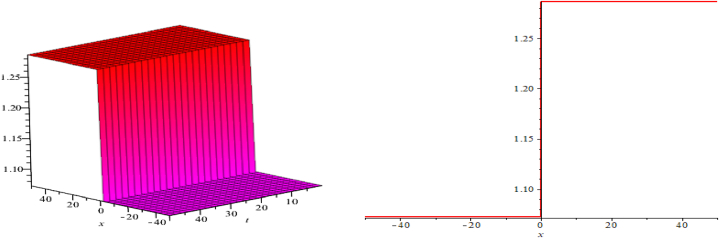
Kink shape soliton, modulus plot of solution ${u_{4}}_{4}$.

**Fig. 5:**
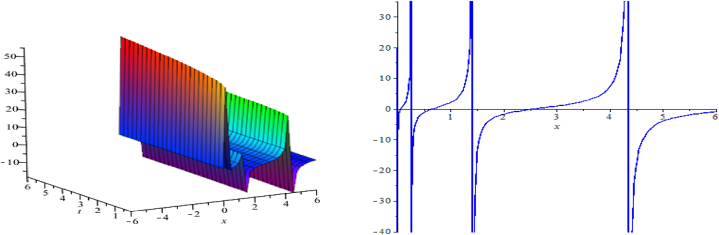
Periodic soliton, modulus plot of solution ${v_{4}}_{5}$.

**Fig. 6:**
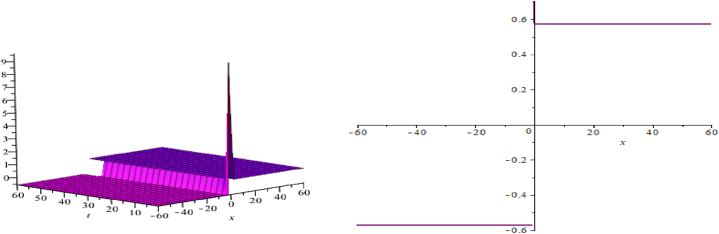
Uneven kink type soliton, modulus plot of solution ${v_{6}}_{4}$.

The wave assessments to the time-space fractional CB equations are numerically simulated in this section. In Fig. 1, Fig. 2, Fig. 3, the solutions attained through the extension of the $\left(G^{\prime }/G\right)$-expansion method are simulated, and the solutions achieved by means of the extension of the generalized $\left(G^{\prime }/G\right)$-expansion method are interpreted in Fig. 4, Fig. 5, Fig. 6. The graphs are depicted by taking appropriate scores of the constraints to make known the contraption of the phenomena modulated by the space-time fractional CB equations. For instance, Fig. 1 shows the shape of soliton depicted from the solution ${u_{1}}_{3}$, for the values λ=2, μ=1, p=−1, q=0.5, d=0, $a_{0}=0$, K=1, $A_{1}=0$, $A_{2}=1$, C=2, $\xi =\frac{10}{3}\left(x^{0.3}+2t^{0.3}\right)$ with fractional order α=0.3; within the limit 0.1≤t≤5, and 0≤x≤5. The modulus plot of ${u_{2}}_{2}$ indicates the singular periodic soliton for μ=0.5, p=−1, q=0.5, λ=−1, d=0, $c_{0}=0$, K=1, $A_{2}=0$, C=1, $\xi =\frac{100}{99}\left(x^{0.99}+t^{0.99}\right)$ and the order of fractional derivative α=0.99 is described in Fig. 2 in the interval 0.01≤t≤0.1 and 0≤x≤20. Solution ${u_{3}}_{1}$ presents the singular kink type soliton for $\lambda =2,\mu =0.5,p=-0.5,q=-0.5,a_{0}=-1,K=3,A_{2}=0,C=3,\xi =\frac{10}{3}\left(x^{0.9}+t^{0.9}\right)$ and the order of fractional derivative α=0.9 is depicted in Fig. 3 in the range −60≤x≤60 and 0.01≤t≤0.1. The solution ${u_{4}}_{4}$ characterizes the kink for A=2, D=1, E=1, $A_{1}=0$, K=9, p=1.1, q=−1, d=5, $a_{0}=0.001$, C=405.38, $\xi =90\left(x^{0.1}+45.04\;t^{0.1}\right)$ and the order of the fractional derivative α=0.1 and plotted in Fig. 4 in the intervals −50≤x≤50 and 0.1≤t≤50. Solution ${v_{4}}_{5}$ indicates the singular periodic soliton for $=2,D=4,E=1,A_{1}=0,K=3,p=-1.1,q=1.1,d=0.001,a_{0}=0.001,C=0.003\text{,}$ $\xi =10\left(x^{0.3}+10^{-3}\;t^{0.3}\right)$, for α=0.3 and outlined in Fig. 5 in the range 0.1≤t≤6 and −6≤x≤6. Solution ${v_{6}}_{4}$ represents the singular kink for λ=2, D=1, E=1, $A_{2}=0$, K=12, p=1.1, q=−1, $a_{0}=0.001$, C=0.5, $\xi =\frac{240}{19}\left(x^{0.95}+0.04\;t^{0.95}\right)$ for fractional-order derivative α=0.95 and sketched in Fig. 6 within the domain 0.1≤t≤60 and −60≤x≤60.

## 6 Validation and advantages

The validity and advantages of proposed extensions over the original $\left(G^{\prime }/G\right)$-expansion scheme and the generalized $\left(G^{\prime }/G\right)$-expansion technique are deliberated in the underneath.

**Validity:** In Ref. [14], Wang et al. suggested the $\left(G^{\prime }/G\right)$-expansion scheme, wherein Eq. (6) is considered as auxiliary equation and exact solution is presented as $u\left(\xi \right)=\sum_{j=0}^{n}a_{j}{\left(G^{\prime }/G\right)}^{j}$, where $a_{n}\neq 0$. Naher and Abdullah [32] used another auxiliary equation (12) and the solutions offered in the shape $u\left(\xi \right)=\sum_{j=0}^{n}a_{j}{\left(d+H\right)}^{j}+\sum_{j=1}^{n}b_{j}{\left(d+H\right)}^{-j}$, where either $a_{n}$ or $a_{-n}$ may be zero. On the other hand, in suggested methods, the form of exact solutions presented as $u\left(\xi \right)=\sum_{j=0}^{n}a_{j}{\left(d+1/\varphi \right)}^{j}+\sum_{j=1}^{n}b_{j}{\left(d+1/\varphi \right)}^{-j}$, where $\varphi \left(\xi \right)=\left(G^{\prime }/G\right)$; and G=G(ξ) satisfies the auxiliary equation (6) in the proposed extension of the $\left(G^{\prime }/G\right)$-expansion method. While in the extension of the generalized $\left(G^{\prime }/G\right)$-expansion method, $\varphi \left(\xi \right)=\left(G^{\prime }/G\right)$; and G=G(ξ) satisfies the auxiliary equation (12).

In [17], Bekir and Güner studied traveling wave solutions of the space-time fractional CB equations by using the original $\left(G^{\prime }/G\right)$-expansion method and constructed only four solutions (A.1a), (A.1b)-(A.4a), (A.4b) (please see Appendix A). On the other hand, by applying the proposed extension of $\left(G^{\prime }/G\right)$-expansion method, we attain nine different solutions. It is significant to perceive that, if we let K=1 and d=0, then the obtained solutions ${u_{2}}_{1}-{u_{2}}_{3}$ and ${v_{2}}_{1}-{v_{2}}_{3}$ are equivalent to the solutions $U_{1}-U_{3}$ and $V_{1}-V_{3}$ (solutions (5.20)–(5.25)) attained by Bekir and Güner [19]. In addition, if we set $A_{2}=0,\lambda >0$ and μ=0, then the attained solutions $u_{2_{1}}$ and $v_{2_{1}}$ are indistinguishable to the solutions (5.26) and (5.27) respectively attained by Bekir and Güner in Ref. [17]. But, if K≠1 and d≠0, the attained solutions ${u_{2}}_{1}-{u_{2}}_{3}$ and ${v_{2}}_{1}-{v_{2}}_{3}$ are different from Bekir and Güner [17] solutions. Moreover, we attain additional solutions. These solutions are novel and were not attained by Bekir and Güner [17].

By using the generalized $\left(G^{\prime }/G\right)$-expansion method, Islam and Akbar [36] obtained ten solutions (solutions (B.1a), (B. 1b)-(B.10a), (B.10b), see appendix B for details) of the space-time fractional CB equations, but by applying the proposed extension of the generalized $\left(G^{\prime }/G\right)$-expansion method we attained twenty solutions. It is noteworthy to point out that, if $A_{1}=0$, then the attained solutions ${u_{6}}_{4}$, ${v_{6}}_{4}$, ${u_{6}}_{5}$ and ${v_{6}}_{5}$ are identical to Islam and Akbar [40] solutions. Thus, the rest of the solutions are new and were not obtained by Islam and Akbar [36].

It is noteworthy to note that the achieved solutions reveal that the proposed methods are robust and efficient in generating many more novel solutions. When comparing the two proposed extensions, it is clear from a comparison of the two extensions that, in terms of performance and insights, the generalized $\left(G^{\prime }/G\right)$-expansion technique outperforms the $\left(G^{\prime }/G\right)$-expansion method.

**Advantages**: The key achievement of projected extensions over the original $\left(G^{\prime }/G\right)$-expansion method and the generalized $\left(G^{\prime }/G\right)$-expansion scheme is that proposed extensions provide further new exact wave solutions and wave solutions of NFEEs have its abundant significance to unveil the internal setup of the tangible phenomena.

## 7 Conclusion

To establish abundant novel and wide-spectral general soliton solutions of NFEE solutions, extensions of the $\left(G^{\prime }/G\right)$-expansion method and the generalized $\left(G^{\prime }/G\right)$-expansion method are presented and implemented in this article. Many new exact general solutions have resulted from these extensions, which provide distinct physical structures with free parameters. In order to illustrate the advantages and validity of the algorithms, we exploit these extensions to the space-time fractional CB equations. To retrieve the well-known and general solutions, we portray the three- and two-dimensional graphs. The structures are capable of explaining the physical phenomena modulated by the space-time fractional CB equations. The obtained results are compared with those accessible in the literature, and it is revealed that these extensions are encouraging mathematical tools compared to existing ones. The results show that the extensions proposed in this article are straightforward, efficient, and applicable to a wide-range of fractional differential equations in mathematical physics.

## Author contribution statement

Altaf A. Al-Shawba: Conceived and designed the experiments; Contributed reagents, materials, analysis tools, or data; Wrote the paper.

Farah A. Abdullah: Performed the experiments; Analyzed and interpreted the data; Contributed reagents, materials, analysis tools, or data.

Amirah Azmi, M. Ali Akbar: Analyzed and interpreted the data; Contributed reagents, materials, analysis tools, or data.

Kottakkaran Sooppy Nisar: Contributed reagents, materials, analysis tools, or data; wrote the paper.

## Data availability statement

No data was used for the research described in the article.

## Declaration of Competing interest

The authors declare that they have no known competing financial interests or personal relationships that could have appeared to influence the work reported in this paper.

## Appendix A Bekir and Güner [17].

Bekir and Güner [17] established solutions of the space-time fractional CB equations by the $\left(G^{\prime }/G\right)$-expansion method which are as follows

When $\rho =\lambda^{2}-4\mu >0$,

(A.1a) $$U_{1}=\frac{b_{0}\left(b-1\right)}{c-1}-\frac{\lambda \left(1-b\right)}{2bc-2}+\frac{\sqrt{\rho }\left(1-b\right)}{2bc-2}\;\left(\frac{B_{1}\;\sinh\left(\frac{\sqrt{\rho }}{2}\;\xi \right){+B}_{2}\;\cosh\left(\frac{\sqrt{\rho }}{2}\;\xi \right)}{B_{1}\;\cosh\left(\frac{\sqrt{\rho }}{2}\;\xi \right)+B_{2}\;\sinh\left(\frac{\sqrt{\rho }}{2}\;\xi \right)}\right)\text{,}$$

(A.1b) $$V_{1}=b_{0}-\frac{\lambda \left(1-c\right)}{2bc-2}+\frac{\sqrt{\rho }\left(1-c\right)}{2bc-2}\;\left(\frac{B_{1}\;\sinh\left(\frac{\sqrt{\rho }}{2}\;\xi \right){+B}_{2}\;\cosh\left(\frac{\sqrt{\rho }}{2}\;\xi \right)}{B_{1}\;\cosh\left(\frac{\sqrt{\rho }}{2}\;\xi \right)+B_{2}\;\sinh\left(\frac{\sqrt{\rho }}{2}\;\xi \right)}\right)\text{,}$$

where $\xi =\frac{t^{\alpha }}{\Gamma \left(1+\alpha \right)}+\frac{2b_{0}-2b_{0}bc-\lambda c+\lambda }{\left(c-1\right)\Gamma \left(1+\alpha \right)}\;t^{\alpha }$.

When $\rho =\lambda^{2}-4\mu <0$,

(A.2a) $$U_{2}=\frac{b_{0}\left(b-1\right)}{c-1}-\frac{\lambda \left(1-b\right)}{2bc-2}+\frac{\sqrt{-\rho }\left(1-b\right)}{2bc-2}\;\left(\frac{{-B}_{1}\;\sin\left(\frac{\sqrt{-\rho }}{2}\;\xi \right){+B}_{2}\;\cos\left(\frac{\sqrt{-\rho }}{2}\;\xi \right)}{B_{1}\;\cos\left(\frac{\sqrt{-\rho }}{2}\;\xi \right)+B_{2}\;\sin\left(\frac{\sqrt{-\rho }}{2}\;\xi \right)}\right)$$

(A.2b) $$V_{2}=b_{0}-\frac{\lambda \left(1-c\right)}{2bc-2}+\frac{\sqrt{-\rho }\left(1-c\right)}{2bc-2}\;\left(\frac{{-B}_{1}\;\sin\left(\frac{\sqrt{-\rho }}{2}\;\xi \right){+B}_{2}\;\cos\left(\frac{\sqrt{-\rho }}{2}\;\xi \right)}{B_{1}\;\cos\left(\frac{\sqrt{-\rho }}{2}\;\xi \right)+B_{2}\;\sin\left(\frac{\sqrt{-\rho }}{2}\;\xi \right)}\right)$$

where $\xi =\frac{t^{\alpha }}{\Gamma \left(1+\alpha \right)}+\frac{2b_{0}-2b_{0}bc-\lambda c+\lambda }{\left(c-1\right)\Gamma \left(1+\alpha \right)}\;t^{\alpha }$.

When $\rho =\lambda^{2}-4\mu =0$,

(A.3a) $$U_{3}=\frac{b_{0}\left(b-1\right)}{c-1}-\frac{\lambda \left(1-b\right)}{2bc-2}+\frac{B_{2}\left(1-b\right)}{\left(bc-1\right)\left(B_{1}+B_{2}\left(\frac{t^{\alpha }}{\Gamma \left(1+\alpha \right)}+\frac{2b_{0}-2b_{0}bc-\lambda c+\lambda }{\left(c-1\right)\Gamma \left(1+\alpha \right)}\;t^{\alpha }\right)\right)}$$

(A.3b) $$V_{3}=b_{0}-\frac{\lambda \left(1-c\right)}{2bc-2}+\frac{B_{2}\left(1-c\right)}{\left(bc-1\right)\left(B_{1}+B_{2}\left(\frac{t^{\alpha }}{\Gamma \left(1+\alpha \right)}+\frac{2b_{0}-2b_{0}bc-\lambda c+\lambda }{\left(c-1\right)\Gamma \left(1+\alpha \right)}\;t^{\alpha }\right)\right)}$$

For $B_{1}\neq 0,B_{2}=0,\lambda >0$ and μ=0, solutions (A.1a) and (A.1b) becomes:

(A.4a) $$u_{1}=\frac{b_{0}\left(b-1\right)}{c-1}-\frac{\lambda \left(1-b\right)}{2bc-2}+\frac{\lambda \left(1-b\right)}{2bc-2}\;\tanh\left(\frac{\lambda }{2}\left(\frac{t^{\alpha }}{\Gamma \left(1+\alpha \right)}+\frac{2b_{0}-2b_{0}bc-\lambda c+\lambda }{\left(c-1\right)\Gamma \left(1+\alpha \right)}\;t^{\alpha }\right)\right)$$

(A.4b) $$v_{1}=b_{0}-\frac{\lambda \left(1-c\right)}{2bc-2}+\frac{\lambda \left(1-c\right)}{2bc-2}\;\tanh\left(\frac{\lambda }{2}\left(\frac{t^{\alpha }}{\Gamma \left(1+\alpha \right)}+\frac{2b_{0}-2b_{0}bc-\lambda c+\lambda }{\left(c-1\right)\Gamma \left(1+\alpha \right)}\;t^{\alpha }\right)\right)$$

### Appendix B Islam and Akbar [36].

Islam and Akbar [36] solved the space-time fractional CB equations by the new generalized $\left(G^{\prime }/G\right)$-expansion method, and they obtained following solutions:

When $B\neq 0,\Omega =B^{2}+4E\psi >0$ and ψ=A−C,

(B.1a) $$u=a_{0}+\frac{m\psi \left(b-1\right)}{A\left(1-bc\right)}\left(P_{1}\right)+\frac{m\left(b-1\right)\left(B^{2}+4E\psi \right)}{4A\psi \left(1-bc\right)}{\left(P_{1}\right)}^{-1}\text{,}$$

(B.1b) $$v=\frac{a_{0}\left(c-1\right)}{b-1}+\frac{m\psi \left(c-1\right)}{A\left(1-bc\right)}\left(P_{1}\right)+\frac{m\left(c-1\right)\left(B^{2}+4E\psi \right)}{4A\psi \left(1-bc\right)}{\left(P_{1}\right)}^{-1}\text{,}$$

where $P_{1}=\frac{\sqrt{\Omega }}{2\psi }\;\frac{B_{1}\;\sinh\left(\frac{\sqrt{\Omega }}{2A}\;\xi \right)+B_{2}\;\cosh\left(\frac{\sqrt{\Omega }}{2A}\;\xi \right)}{B_{1}\;\cosh\left(\frac{\sqrt{\Omega }}{2A}\;\xi \right)+B_{2}\;\sinh\left(\frac{\sqrt{\Omega }}{2A}\;\xi \right)}$, $\xi =\left(x-\frac{Ct^{\alpha }}{\Gamma \left(1+\alpha \right)}\right)$ and $B_{1},B_{2}$ are arbitrary constants. For $B_{1}=0$ but $B_{2}\neq 0$, solutions (B.1a) and (B.1b) turn into

(B.2a) $$u_{1}=a_{0}+\frac{m\psi \left(b-1\right)}{A\left(1-bc\right)}\left(P_{2}\right)+\frac{m\left(b-1\right)\left(B^{2}+4E\psi \right)}{4A\psi \left(1-bc\right)}{\left(P_{2}\right)}^{-1}\text{,}$$

(B.2b) $$v_{1}=\frac{a_{0}\left(c-1\right)}{b-1}+\frac{m\psi \left(c-1\right)}{A\left(1-bc\right)}\left(P_{2}\right)+\frac{m\left(c-1\right)\left(B^{2}+4E\psi \right)}{4A\psi \left(1-bc\right)}{\left(P_{2}\right)}^{-1}\text{,}$$

where $P_{2}=\frac{B_{2}\sqrt{\Omega }}{2\psi }\;\coth\left(\frac{\sqrt{\Omega }}{2A}k\left(x-\frac{Ct^{\alpha }}{\Gamma \left(1+\alpha \right)}\right)\right)$. For $B_{2}=0$ but $B_{1}\neq 0$, then the solutions (B.1a) and (B.1b) are simplified as:

(B.3a) $$u_{2}=a_{0}+\frac{m\psi \left(b-1\right)}{A\left(1-bc\right)}\left(P_{3}\right)+\frac{m\left(b-1\right)\left(B^{2}+4E\psi \right)}{4A\psi \left(1-bc\right)}{\left(P_{3}\right)}^{-1}\text{,}$$

(B.3b) $$v_{2}=\frac{a_{0}\left(c-1\right)}{b-1}+\frac{m\psi \left(c-1\right)}{A\left(1-bc\right)}\left(P_{3}\right)+\frac{m\left(c-1\right)\left(B^{2}+4E\psi \right)}{4A\psi \left(1-bc\right)}{\left(P_{3}\right)}^{-1}\text{,}$$

where $P_{3}=\frac{B_{1}\sqrt{\Omega }}{2\psi }\;\tanh\left(\frac{\sqrt{\Omega }}{2A}k\left(x-\frac{Ct^{\alpha }}{\Gamma \left(1+\alpha \right)}\right)\right)$.

When $B\neq 0,\Omega =B^{2}+4E\psi <0,\psi =A-C$ and if $B_{1}=0$ but $B_{2}\neq 0$, then they obtained the traveling wave solutions:

(B.4a) $$u_{3}=a_{0}+\frac{m\psi \left(b-1\right)}{A\left(1-bc\right)}\left(P_{4}\right)+\frac{m\left(b-1\right)\left(B^{2}+4E\psi \right)}{4A\psi \left(1-bc\right)}{\left(P_{4}\right)}^{-1}\text{,}$$

(B.4b) $$v_{3}=\frac{a_{0}\left(c-1\right)}{b-1}+\frac{m\psi \left(c-1\right)}{A\left(1-bc\right)}\left(P_{4}\right)+\frac{m\left(c-1\right)\left(B^{2}+4E\psi \right)}{4A\psi \left(1-bc\right)}{\left(P_{4}\right)}^{-1}\text{,}$$

where $P_{4}=\frac{B_{2}\sqrt{-\Omega }}{2\psi }\;\cot\left(\frac{\sqrt{-\Omega }}{2A}k\left(x-\frac{Ct^{\alpha }}{\Gamma \left(1+\alpha \right)}\right)\right)$. For $B_{2}=0$ but $B_{1}\neq 0$, then they obtained the exact solutions:

(B.5a) $$u_{4}=a_{0}+\frac{m\psi \left(b-1\right)}{A\left(1-bc\right)}\left(P_{5}\right)+\frac{m\left(b-1\right)\left(B^{2}+4E\psi \right)}{4A\psi \left(1-bc\right)}{\left(P_{5}\right)}^{-1}\text{,}$$

(B.5b) $$v_{4}=\frac{a_{0}\left(c-1\right)}{b-1}+\frac{m\psi \left(c-1\right)}{A\left(1-bc\right)}\left(P_{5}\right)+\frac{m\left(c-1\right)\left(B^{2}+4E\psi \right)}{4A\psi \left(1-bc\right)}{\left(P_{5}\right)}^{-1}\text{,}$$

where $P_{5}=-\frac{B_{1}\sqrt{-\Omega }}{2\psi }\;\tan\left(\frac{\sqrt{-\Omega }}{2A}k\left(x-\frac{Ct^{\alpha }}{\Gamma \left(1+\alpha \right)}\right)\right)$.

When $B\neq 0,\Omega =B^{2}+4E\psi =0,\psi =A-C$ and if $B_{1}=0$ but $B_{2}\neq 0$, then they obtained the following solutions:

(B.6a) $$u_{5}=a_{0}+\frac{m\psi \left(b-1\right)}{A\left(1-bc\right)}\left(\frac{1}{k}{\left(x-\frac{Ct^{\alpha }}{\Gamma \left(1+\alpha \right)}\right)}^{-1}\right)+\frac{m\left(b-1\right)\left(B^{2}+4E\psi \right)}{4A\psi \left(1-bc\right)}{\left(\frac{1}{k}{\left(x-\frac{Ct^{\alpha }}{\Gamma \left(1+\alpha \right)}\right)}^{-1}\right)}^{-1}\text{,}$$

(B.6b) $$v_{5}=\frac{a_{0}\left(c-1\right)}{b-1}+\frac{m\psi \left(c-1\right)}{A\left(1-bc\right)}\left(\frac{1}{k}{\left(x-\frac{Ct^{\alpha }}{\Gamma \left(1+\alpha \right)}\right)}^{-1}\right)+\frac{m\left(c-1\right)\left(B^{2}+4E\psi \right)}{4A\psi \left(1-bc\right)}{\left(\frac{1}{k}{\left(x-\frac{Ct^{\alpha }}{\Gamma \left(1+\alpha \right)}\right)}^{-1}\right)}^{-1}\text{.}$$

When B=0,Δ=Eψ>0,ψ=A−C and if $B_{1}=0$ but $B_{2}\neq 0$, then they obtained the traveling wave solutions:

(B.7a) $$u_{6}=a_{0}+\frac{m\psi \left(b-1\right)}{A\left(1-bc\right)}\left(P_{6}\right)+\frac{m\left(b-1\right)\left(B^{2}+4E\psi \right)}{4A\psi \left(1-bc\right)}{\left(P_{6}\right)}^{-1}$$

(B.7b) $$v_{6}=\frac{a_{0}\left(c-1\right)}{b-1}+\frac{m\psi \left(c-1\right)}{A\left(1-bc\right)}\left(P_{6}\right)+\frac{m\left(c-1\right)\left(B^{2}+4E\psi \right)}{4A\psi \left(1-bc\right)}{\left(P_{6}\right)}^{-1}$$

where $P_{6}=-\frac{B}{2\psi }+\frac{B_{2}\sqrt{\Delta }}{\psi }\;\coth\left(\frac{\sqrt{\Delta }}{A}k\left(x-\frac{Ct^{\alpha }}{\Gamma \left(1+\alpha \right)}\right)\right)$. For $B_{2}=0$ but $B_{1}\neq 0$, then they obtained the exact wave solutions:

(B.8a) $$u_{7}=a_{0}+\frac{m\psi \left(b-1\right)}{A\left(1-bc\right)}\left(P_{7}\right)+\frac{m\left(b-1\right)\left(B^{2}+4E\psi \right)}{4A\psi \left(1-bc\right)}{\left(P_{7}\right)}^{-1}$$

(B.8b) $$v_{7}=\frac{a_{0}\left(c-1\right)}{b-1}+\frac{m\psi \left(c-1\right)}{A\left(1-bc\right)}\left(P_{7}\right)+\frac{m\left(c-1\right)\left(B^{2}+4E\psi \right)}{4A\psi \left(1-bc\right)}{\left(P_{7}\right)}^{-1}\text{,}$$

where $P_{7}=-\frac{B}{2\psi }+\frac{B_{1}\sqrt{\Delta }}{\psi }\;\tanh\left(\frac{\sqrt{\Delta }}{A}k\left(x-\frac{Ct^{\alpha }}{\Gamma \left(1+\alpha \right)}\right)\right)$.

When B=0,Δ=Eψ<0,ψ=A−C and if $B_{1}=0$ but $B_{2}\neq 0$, then they obtained the following solutions:

(B.9a) $$u_{8}=a_{0}+\frac{m\psi \left(b-1\right)}{A\left(1-bc\right)}\left(P_{8}\right)+\frac{m\left(b-1\right)\left(B^{2}+4E\psi \right)}{4A\psi \left(1-bc\right)}{\left(P_{8}\right)}^{-1}\text{,}$$

(B.9b) $$v_{8}=\frac{a_{0}\left(c-1\right)}{b-1}+\frac{m\psi \left(c-1\right)}{A\left(1-bc\right)}\left(P_{8}\right)+\frac{m\left(c-1\right)\left(B^{2}+4E\psi \right)}{4A\psi \left(1-bc\right)}{\left(P_{8}\right)}^{-1}\text{,}$$

where $P_{8}=-\frac{B}{2\psi }+\frac{B_{2}\sqrt{-\Delta }}{\psi }\;\cot\left(\frac{\sqrt{-\Delta }}{A}k\left(x-\frac{Ct^{\alpha }}{\Gamma \left(1+\alpha \right)}\right)\right)$. For $B_{2}=0$ but $B_{1}\neq 0$, then they obtained the exact wave solutions:

(B.10a) $$u_{9}=a_{0}+\frac{m\psi \left(b-1\right)}{A\left(1-bc\right)}\left(P_{9}\right)+\frac{m\left(b-1\right)\left(B^{2}+4E\psi \right)}{4A\psi \left(1-bc\right)}{\left(P_{9}\right)}^{-1}\text{,}$$

(B.10b) $$v_{9}=\frac{a_{0}\left(c-1\right)}{b-1}+\frac{m\psi \left(c-1\right)}{A\left(1-bc\right)}\left(P_{9}\right)+\frac{m\left(c-1\right)\left(B^{2}+4E\psi \right)}{4A\psi \left(1-bc\right)}{\left(P_{9}\right)}^{-1}\text{,}$$

where $P_{9}=-\frac{B}{2\psi }-\frac{B_{1}\sqrt{-\Delta }}{\psi }\;\tan\left(\frac{\sqrt{-\Delta }}{A}k\left(x-\frac{Ct^{\alpha }}{\Gamma \left(1+\alpha \right)}\right)\right)$ and $k=\frac{2ma_{0}\left(1-bc\right)}{b-1}$.
